# Single-nucleus transcriptomics decodes the link between aging and lumbar disc herniation

**DOI:** 10.1093/procel/pwaf025

**Published:** 2025-03-22

**Authors:** Min Wang, Zan He, Anqi Wang, Shuhui Sun, Jiaming Li, Feifei Liu, Chunde Li, Chengxian Yang, Jinghui Lei, Yan Yu, Shuai Ma, Si Wang, Weiqi Zhang, Zhengrong Yu, Guang-Hui Liu, Jing Qu

**Affiliations:** State Key Laboratory of Organ Regeneration and Reconstruction, Institute of Zoology, Chinese Academy of Sciences, Beijing 100101, China; Division of Life Sciences and Medicine, School of Life Sciences, University of Science and Technology of China, Hefei 230001, China; Advanced Innovation Center for Human Brain Protection, National Clinical Research Center for Geriatric Disorders, Xuanwu Hospital, Capital Medical University, Beijing 100053, China; Aging Translational Medicine Center, Beijing Municipal Geriatric Medical Research Center, Xuanwu Hospital, Capital Medical University, Beijing 100053, China; Department of Orthopaedics, Peking University First Hospital, Beijing 100034, China; Department of Musculoskeletal Oncology, Sun Yat-sen University Cancer Center, State Key Laboratory of Oncology in South China, Collaborative Innovation Center for Cancer Medicine, Guangzhou 510060, China; State Key Laboratory of Organ Regeneration and Reconstruction, Institute of Zoology, Chinese Academy of Sciences, Beijing 100101, China; University of Chinese Academy of Sciences, Beijing 100049, China; Beijing Institute for Stem Cell and Regenerative Medicine, Beijing 100101, China; Beijing Institute of Heart Lung and Blood Vessel Diseases, Beijing Anzhen Hospital, Capital Medical University, Beijing 100029, China; University of Chinese Academy of Sciences, Beijing 100049, China; China National Center for Bioinformation, Beijing 100101, China; Beijing Institute of Genomics, Chinese Academy of Sciences, Beijing 100101, China; Beijing Institute of Heart Lung and Blood Vessel Diseases, Beijing Anzhen Hospital, Capital Medical University, Beijing 100029, China; Department of Orthopaedics, Peking University First Hospital, Beijing 100034, China; Department of Orthopaedics, Peking University First Hospital, Beijing 100034, China; Advanced Innovation Center for Human Brain Protection, National Clinical Research Center for Geriatric Disorders, Xuanwu Hospital, Capital Medical University, Beijing 100053, China; Aging Translational Medicine Center, Beijing Municipal Geriatric Medical Research Center, Xuanwu Hospital, Capital Medical University, Beijing 100053, China; State Key Laboratory of Organ Regeneration and Reconstruction, Institute of Zoology, Chinese Academy of Sciences, Beijing 100101, China; University of Chinese Academy of Sciences, Beijing 100049, China; Beijing Institute for Stem Cell and Regenerative Medicine, Beijing 100101, China; State Key Laboratory of Organ Regeneration and Reconstruction, Institute of Zoology, Chinese Academy of Sciences, Beijing 100101, China; University of Chinese Academy of Sciences, Beijing 100049, China; Beijing Institute for Stem Cell and Regenerative Medicine, Beijing 100101, China; Aging Biomarker Consortium, Beijing 100101, China; Advanced Innovation Center for Human Brain Protection, National Clinical Research Center for Geriatric Disorders, Xuanwu Hospital, Capital Medical University, Beijing 100053, China; Aging Translational Medicine Center, Beijing Municipal Geriatric Medical Research Center, Xuanwu Hospital, Capital Medical University, Beijing 100053, China; Aging Biomarker Consortium, Beijing 100101, China; University of Chinese Academy of Sciences, Beijing 100049, China; China National Center for Bioinformation, Beijing 100101, China; Beijing Institute of Genomics, Chinese Academy of Sciences, Beijing 100101, China; Aging Biomarker Consortium, Beijing 100101, China; Department of Orthopaedics, Peking University First Hospital, Beijing 100034, China; State Key Laboratory of Organ Regeneration and Reconstruction, Institute of Zoology, Chinese Academy of Sciences, Beijing 100101, China; Advanced Innovation Center for Human Brain Protection, National Clinical Research Center for Geriatric Disorders, Xuanwu Hospital, Capital Medical University, Beijing 100053, China; Aging Translational Medicine Center, Beijing Municipal Geriatric Medical Research Center, Xuanwu Hospital, Capital Medical University, Beijing 100053, China; University of Chinese Academy of Sciences, Beijing 100049, China; Beijing Institute for Stem Cell and Regenerative Medicine, Beijing 100101, China; Aging Biomarker Consortium, Beijing 100101, China; State Key Laboratory of Organ Regeneration and Reconstruction, Institute of Zoology, Chinese Academy of Sciences, Beijing 100101, China; University of Chinese Academy of Sciences, Beijing 100049, China; Beijing Institute for Stem Cell and Regenerative Medicine, Beijing 100101, China; Beijing Institute of Heart Lung and Blood Vessel Diseases, Beijing Anzhen Hospital, Capital Medical University, Beijing 100029, China; Aging Biomarker Consortium, Beijing 100101, China

**Keywords:** aging, herniation, nucleus pulposus, single-nucleus transcriptomics, NFAT1, senescence

## Abstract

Lumbar disc (LD) herniation and aging are prevalent conditions that can result in substantial morbidity. This study aimed to clarify the mechanisms connecting the LD aging and herniation, particularly focusing on cellular senescence and molecular alterations in the nucleus pulposus (NP). We performed a detailed analysis of NP samples from a diverse cohort, including individuals of varying ages and those with diagnosed LD herniation. Our methodology combined histological assessments with single-nucleus RNA sequencing to identify phenotypic and molecular changes related to NP aging and herniation. We discovered that cellular senescence and a decrease in nucleus pulposus progenitor cells (NPPCs) are central to both processes. Additionally, we found an age-related increase in NFAT1 expression that promotes NPPC senescence and contributes to both aging and herniation of LD. This research offers fresh insights into LD aging and its associated pathologies, potentially guiding the development of new therapeutic strategies to target the root causes of LD herniation and aging.

## Introduction

Low back pain, predominantly caused by lumbar disc (LD) herniation, is the most significant contributor to global disability and its prevalence increases with aging (Chou, 2021; Hartvigsen et al., 2018; Ye et al., 2022; Zhang et al., 2023a). The LD, a cartilaginous structure that connects adjacent vertebral bodies, is composed of the gel-like nucleus pulposus (NP), the annulus fibrosus, and the outer cartilage end plates (Mohd Isa et al., 2022; Roberts et al., 2006). It provides flexibility to the vertebral column, maintaining a deformable space between intervertebral bodies and acting as a shock absorber under compressive loads (Humzah and Soames, 1988; Raj, 2008). The NP, rich in extracellular matrix (ECM) and hosting nucleus pulposus progenitor cells (NPPCs), is crucial for the differentiation into chondrocytes and the overall homeostasis of the LD (Chen et al., 2017; Choi et al., 2015; Lyu et al., 2019).

LD herniation is characterized by the protrusion of the NP, which can irritate and compress nerve roots and the cauda equina, leading to severe lumbar pain (Wong et al., 2023; Zhang et al., 2023a). This condition not only compromises quality of life by causing chronic pain and potential functional impairment but also imposes substantial socioeconomic burdens (Amin et al., 2017; Chou, 2021; Deyo and Mirza, 2016; Hartvigsen et al., 2018). Studies have shown a positive correlation between the incidence of LD herniation and age (Boden et al., 1990; Dammers and Koehler, 2002; Ito et al., 2023), suggesting that the age-related degeneration of LD may increase its vulnerability to structural failure (Hwang et al., 2014; Raj, 2008; Roughley et al., 2002; Vo et al., 2016). However, the detailed relationship between aging and LD herniation, as well as the underlying pathophysiological mechanisms, is not fully understood and warrants further exploration.

In this study, we aimed to investigate the phenotypic and molecular parallels and divergences between LD aging and herniation using histological examination and single-nucleus RNA sequencing of human NP samples. We identified similarities in ECM degradation and senescent cell accumulation between aging and herniated NP tissues, with a notable loss of NPPCs as a common feature. We discovered that the expression of the transcription factor NFAT1 (encoded by *NFATC2*) was upregulated in NPPCs from both aged and herniated individuals. Furthermore, activation of *NFATC2* accelerated NPPC senescence, while its knockout delayed the process. These findings point to a shared underlying mechanism for LD aging and herniation and position *NFATC2* as a potential therapeutic target for interventions in LD aging and related disorders.

## Results

### Phenotypic characteristics of the NP in aged and herniated LDs

**Figure 1.**
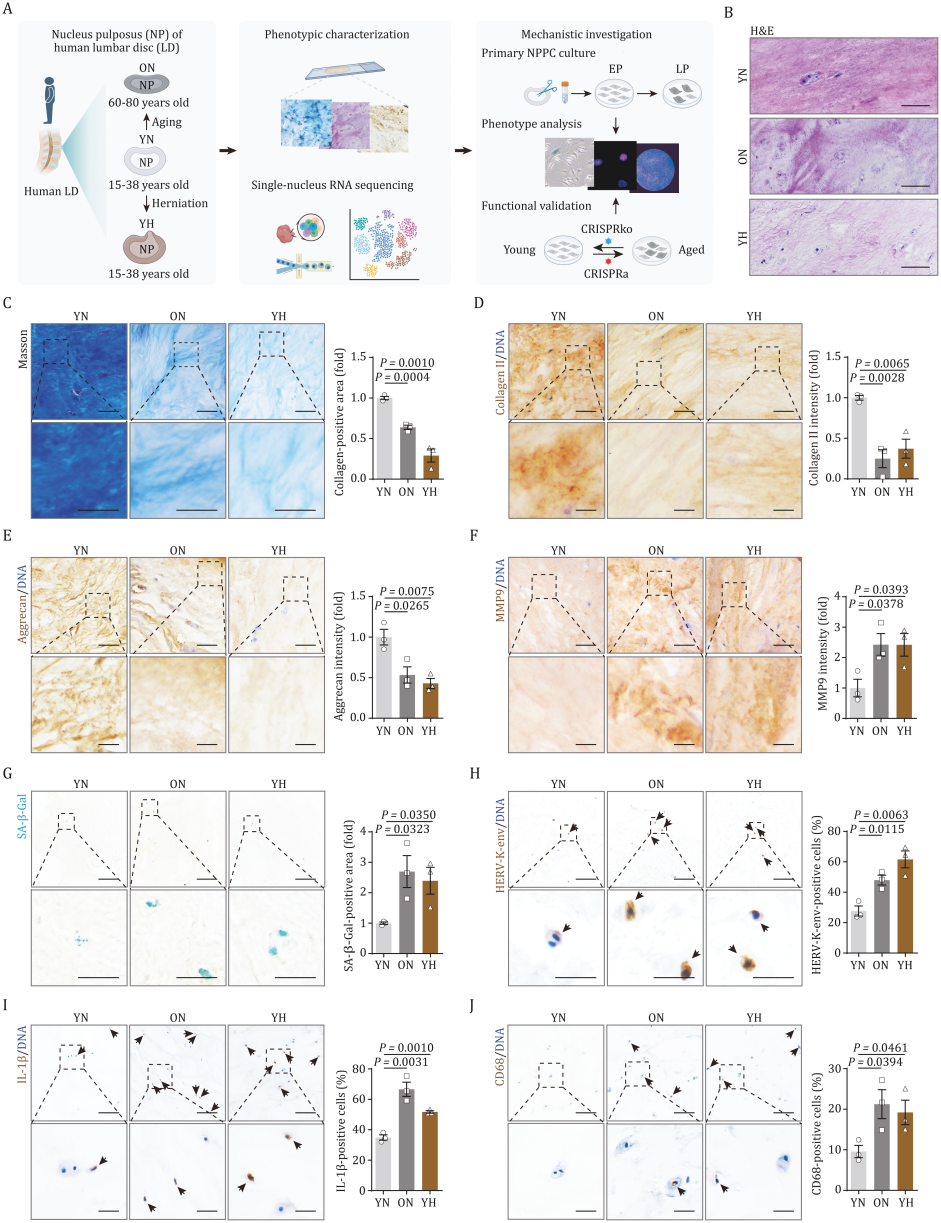
**Phenotypic alterations in NP of aged and herniated human LDs.** (A) Schematic diagram of the experimental design for depicting cellular and molecular regulatory network involved in human NP aging and herniation. YN, young NP; ON, old NP; YH, young herniated NP. (B) H&E staining in human NP sections of YN, ON and YH. Scale bars, 100 μm. (C) Masson-trichrome staining in human NP sections of YN, ON and YH. Left, representative images, scale bars, 50 μm and 25 μm (zoomed-in images). Right, the collagen-positive area is quantified as fold change. (D) Immunohistochemistry staining for collagen II in human NP sections of YN, ON and YH. Left, representative images, scale bars, 100 μm and 25 μm (zoomed-in images). Right, the intensity of collagen II is quantified as fold change. (E) Immunohistochemistry staining for aggrecan in human NP sections of YN, ON and YH. Left, representative images, scale bars, 100 μm and 25 μm (zoomed-in images). Right, the intensity of aggrecan is quantified as fold change. (F) Immunohistochemistry staining for MMP9 in human NP sections of YN, ON and YH. Left, representative images, scale bars, 100 μm and 25 μm (zoomed-in images). Right, the intensity of MMP9 is quantified as fold change. (G) SA-β-Gal staining in human NP sections of YN, ON and YH. Left, representative images, scale bars, 100 μm and 25 μm (zoomed-in images). Right, SA-β-Gal-positive area is quantified as fold change. (H) Immunohistochemistry staining for HERV-K-env in human NP sections of YN, ON and YH. Left, representative images, scale bars, 100 μm and 25 μm (zoomed-in images). Right, the proportion of HERV-K-env-positive cells per field of vision is quantified. The arrows indicate HERV-K-env-positive cells. (I) Immunohistochemistry staining for IL-1β in human NP sections of YN, ON and YH. Left, representative images, scale bars, 100 μm and 25 μm (zoomed-in images). Right, the proportion of IL-1β-positive cells per field of vision is quantified. The arrows indicate IL-1β-positive cells. (J) Immunohistochemistry staining for CD68 in human NP sections of YN, ON and YH. Left, representative images, scale bars, 100 μm and 25 μm (zoomed-in images). Right, the proportion of CD68-positive cells per field of vision is quantified. The arrows indicate CD68-positive cells. Data are presented as the mean ± SEM. *n* = 3 individuals per group. Statistical significance was assessed using two-tailed Student’s unpaired *t*-tests. The figure 1A was created with BioRender.com.

To explore the phenotypic changes in the NP associated with aging and herniation, we collected human lumbar disc NP samples and categorized them into three groups: young NP (YN), old NP (ON), and young herniated NP (YH) for analysis (Fig. 1A). Histological examination using H&E and Masson staining revealed a disorganized NP structure in both ON and YH groups compared to the YN group, alongside a reduction in total collagen, the primary ECM proteins in NP tissue (Fig. 1B and 1C). This was further supported by immunohistochemical staining for collagen II, the predominant collagen type in NP, and aggrecan, the major proteoglycan, which both showed a decrease in ON and YH groups (Fig. 1D and 1E). In line with ECM loss, the expression of MMP9, a matrix remodeling enzyme involved in the degradation of collagen II and aggrecan, was upregulated in both ON and YH groups compared to the YN group (Fig. 1F). These observations indicate that both aged and herniated NP tissues display similar structural degeneration marked by ECM dysregulation.

The accumulation of senescent cells, a hallmark of aging implicated in various diseases (Aging Biomarker Consortium et al., 2023b; Cai et al., 2022; Chen and Tang, 2019; Jing et al., 2023; Li et al., 2024a; Lu et al., 2024; Sun et al., 2023; Wang et al., 2016; Wu et al., 2024a; Yang et al., 2024a; Ye et al., 2024; Zhang et al., 2021, 2023b), is understudied in the NP. We detected an increased presence of senescence-associated β-galactosidase (SA-β-Gal)-positive area and a higher proportion of p21^Cip1^-positive cells in both ON and YH groups (Figs. 1G and S1A). Meantime, the Ki67-positive cells were decreased (Fig. S1B). Additionally, the expression of human endogenous retrovirus-K (HERV-K), a novel senescence marker (Liu et al., 2023; Wu et al., 2024b), was elevated in both ON and YH groups (Fig. 1H). Senescent cells are characterized by the secretion of inflammatory factors and chemokines, known collectively as the senescence-associated secretory phenotype (SASP) (Aging Biomarker Consortium et al., 2023a; Li et al., 2024b; Lopez-Otin et al., 2023; Yang et al., 2024c; Zhang et al., 2024). We found that interleukin-1β (IL-1β), a prototypical SASP and pro-inflammatory cytokine, was upregulated in ON and YH groups (Fig. 1I). Furthermore, the infiltration of immune cells, including CD68-positive macrophages and CD45-positive immune cells, was increased in ON and YH groups (Figs. 1J and S1C). In conclusion, our histological analysis disclosed analogous changes in structural disorganization and cellular senescence in both herniated and aged NP tissues.

### Single-nucleus transcriptomic dissection of aged and herniated NP tissues

**Figure 2.**
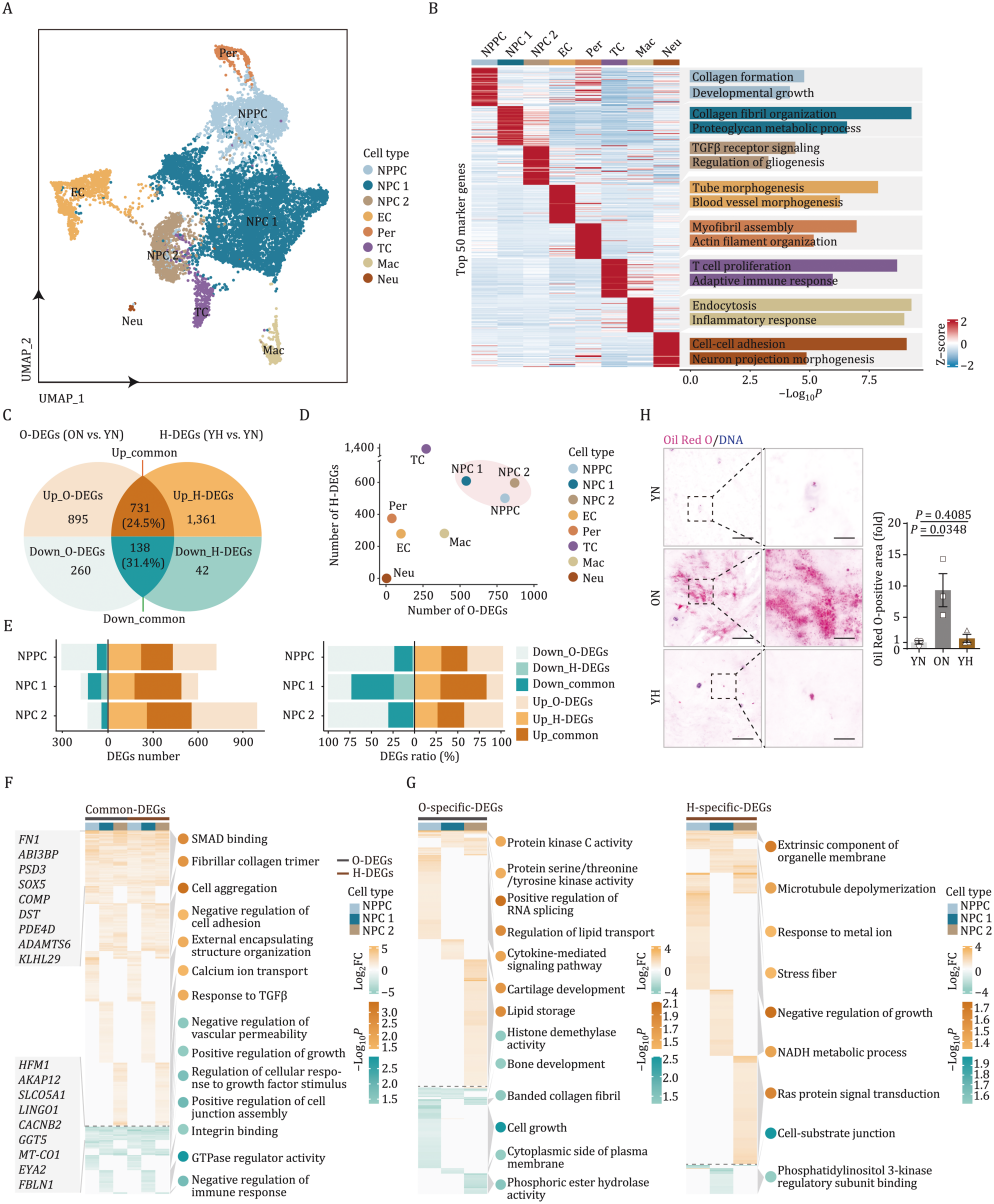
**Single-nucleus transcriptomic profiling of aged and herniated human NPs.** (A) UMAP plot showing the distribution of cell types in human NP. NPPC, nucleus pulposus progenitor cell; NPC 1, nucleus pulposus cell 1; NPC 2, nucleus pulposus cell 2; EC, endothelial cell; Per, pericyte; TC, T cell; Mac, macrophage; Neu, neuron-like cell. (B) Heatmap showing the expression profiles of the top 50 cell type-specific marker genes for each cell type in human NP, with corresponding functional annotations on the right. (C) Venn diagram showing the DEGs number of human old NP (O-DEGs) and herniated NP (H-DEGs), the regions that intersect represent common DEGs and the regions that do not intersect represent specific DEGs for each group. (D) Dotplot showing the DEGs number of O-DEGs and H-DEGs in all cell types. (E) Barplots showing the common and specific DEGs number (left) or ratio (right) of chondrocytes in human aged and herniated NP. Chondrocytes include NPPC, NPC 1, and NPC 2. (F) Heatmap showing the common DEGs of O-DEGs and H-DEGs in chondrocytes in human NP. The color key represents scaled log_2_FC of DEGs. FC, fold change. Dotplots illustrating GO enrichment analysis of cell type specific DEGs in chondrocyte populations. The color of dots represents −log_10_*P* of enrichment. (G) Heatmap showing the O-specific DEGs (left) and the H-specific DEGs (right) of chondrocytes in human NP. The color key represents scaled log_2_FC of DEGs. Dotplots illustrating GO enrichment analysis of cell type specific DEGs in chondrocytes populations. The color of dots represents −log_10_*P* of enrichment. (H) Oil Red O staining in human NP sections of YN, ON and YH. Left, representative images. Scale bars, 100 μm and 25 μm (zoomed-in images). Right, Oil Red O-positive area is quantified as fold change. Data are presented as the mean ± SEM. *n* = 3 individuals per group. Statistical significance was assessed using two-tailed Student’s unpaired *t*-tests.

To dissect the molecular underpinnings of aging and herniation in the NP, we constructed a single-nucleus transcriptomic atlas from NP tissues across YN, ON, and YH groups. After quality control, we obtained 10,884 single-nucleus transcriptomes, which were clustered into eight distinct cell types based on marker genes: NPPC, nucleus pulposus cell 1 (NPC 1), nucleus pulposus cell 2 (NPC 2), endothelial cell (EC), pericyte (Per), T cell (TC), macrophage (Mac), and neuron-like cell (Neu) (Figs. 2A and S2A; Table S2). Pathway enrichment analysis of the top 50 marker genes for each cell type highlighted their functional roles, such as “collagen formation” and “developmental growth” for NPPC and “T cell proliferation” for TC (Fig. 2B).

Differentially expressed genes (DEGs) analysis between groups identified 2,024 DEGs in ON versus YN (O-DEGs), with 1,626 upregulated and 398 downregulated genes, and 2,272 DEGs in YH versus YN (H-DEGs), with 2,092 upregulated and 180 downregulated genes (Log_2_FC ≥ 0.75, *P*.adjust < 0.05) (Fig. 2C; Table S3). A significant overlap of 731 upregulated and 138 downregulated genes was observed between aging and herniation, indicating substantial similarities in gene expression patterns (Fig. 2C). Chondrocytes, including NPPC and NPCs, the primary cellular components of NP tissues, showed the most pronounced gene expression changes during aging and herniation (Fig. 2D), underscoring their role in NP homeostasis and directing our focus toward NPCs and NPPCs.

DEGs analysis within chondrocyte populations revealed a predominance of upregulated genes, with approximately one-quarter shared between ON and YH cells (Fig. 2E). *FN1* and *COMP*, known to function synergistically as ECM components in the NP (Bhattarai et al., 2024; Cherif et al., 2022; Flowers et al., 2017; Lv et al., 2016; Yang et al., 2009), were consistently upregulated across chondrocyte types (Fig. 2F). Conversely, *FBLN1*, which promotes chondrocyte proliferation and collagen expression (Xu et al., 2022), was downregulated in both ON and YH groups (Fig. 2F). Gene Ontology (GO) term analysis of DEGs in chondrocytes revealed common upregulation of genes associated with cell aggregation and TGFβ/SMAD signaling in NPC 1 and NPC 2, suggesting a role in LD degeneration and ossification (Lama et al., 2019) (Fig. 2F). DEG analysis also revealed distinct molecular signatures between conditions, with ON samples showing upregulation of lipid storage-associated genes while YH samples exhibited increased expression of stress fiber-related genes (Fig. 2G). Consistent with these findings, Oil Red O staining demonstrated selective accumulation of lipid droplets in ON samples (Fig. 2H).

In summary, our single-nucleus transcriptomic profiling of human NP samples delineated cell type-specific gene expression changes, highlighting both shared and unique molecular responses of chondrocytes to LD aging and herniation.

### Exhaustion of NPPCs in aged and herniated NP tissues

**Figure 3.**
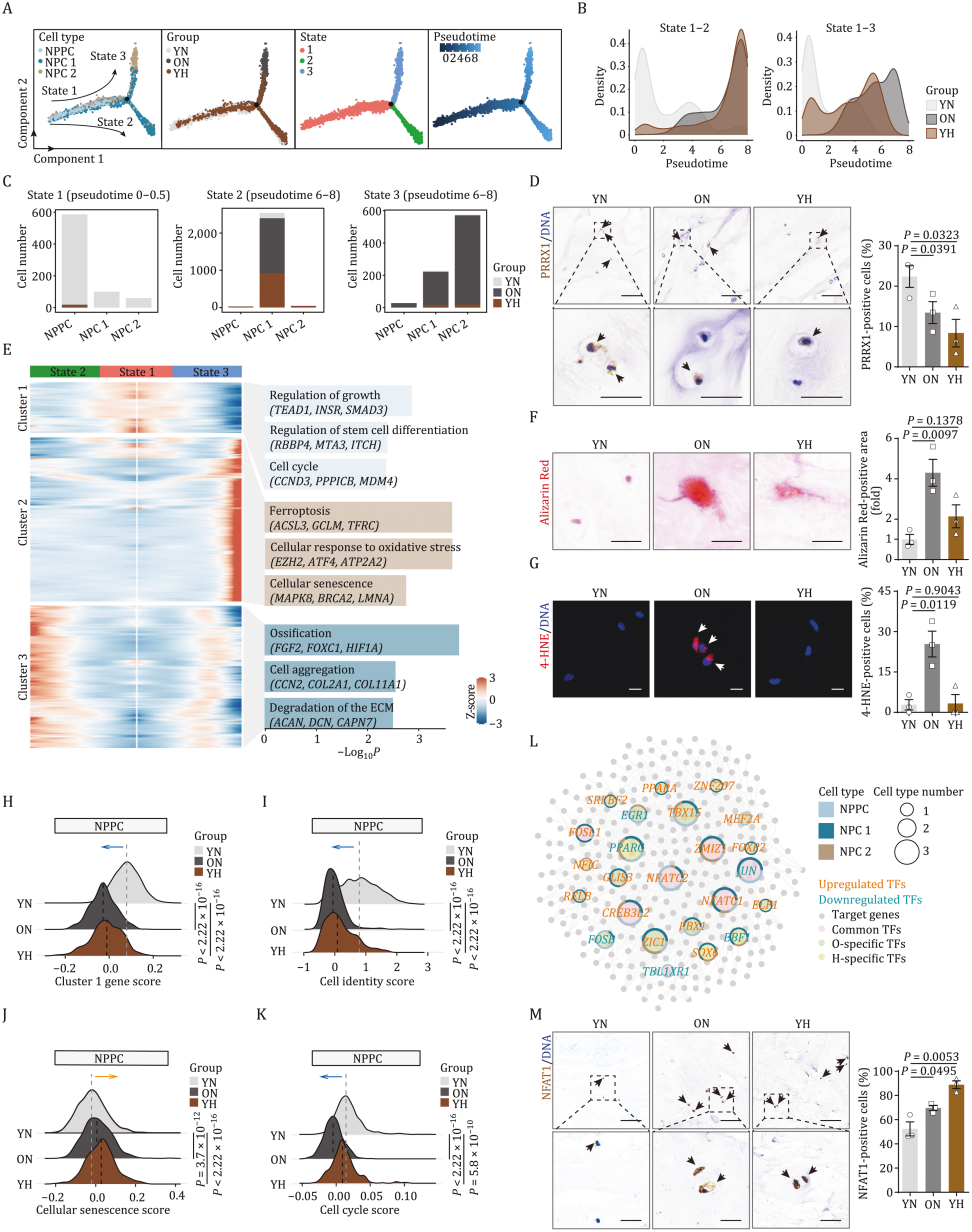
**Elevated NFAT1 expression and NPPC senescence in aged and herniated NP.** (A) Pseudotime trajectory showing the cell arrangement of NPPC and NPCs. The color of the cells in four figures from left to right represents the state of cell types, sample source, pseudotime state and pseudotime value, respectively. (B) Density plots showing the alteration in sample group density of chondrocytes from state 1 to state 2 or state 1 to state 3, respectively. (C) Barplots showing the cell type and cell number at early (pseudotime 0–0.5) and late (pseudotime 6–8) stages of pseudotime. (D) Immunohistochemistry staining of PRRX1 in human NP sections of YN, ON and YH. Left, representative images, scale bars, 100 μm and 25 μm (zoomed-in images). Right, the proportion of PRRX1-positive cells is quantified. The arrows indicate PRRX1-positive cells. (E) Scaled expression heatmap of genes changing with pseudotime. Genes were divided into three clusters according to their different changing patterns, and the genes in each cluster were analyzed for GO enrichment analysis. (F) Alizarin Red staining in human NP sections of YN, ON and YH. Left, representative images, scale bars, 25 μm. Right, the Alizarin Red-positive area is quantified as fold change. (G) Immunofluorescence staining of 4-HNE in human NP sections of YN, ON and YH. Left, representative images, scale bars, 10 μm. Right, the proportion of 4-HNE-positive cells is quantified. The arrows indicate 4-HNE-positive cells. (H) Score analysis for the expression of cluster 1 genes in NPPC in human NP of YN, ON and YH. (I) Score analysis for the expression of cell identity genes in NPPC in human NP of YN, ON and YH. (J) Score analysis for the expression of cellular senescence related gene sets in NPPC of human NP of YN, ON and YH. (K) Score analysis for the expression of cell cycle related gene sets in NPPC of human NP of YN, ON and YH. (L) Transcription factors (TFs) regulatory network showing the common and specific TFs which were differentially expressed in ON or YH compared to YN. The size of the dots represents the cell types in which the TFs were expressed, and the cell types were labeled by loops. The gray points represent the target genes of TFs. (M) Immunohistochemistry staining of NFAT1 in human NP sections of YN, ON and YH. Left, representative images, scale bars, 100 μm and 25 μm (zoomed-in images). Right, the proportion of NFAT1-positive cells is quantified. The arrows indicate NFAT1-positive cells. Data are presented as the mean ± SEM. *n* = 3 individuals per group. Statistical significance was assessed using two-tailed Student’s unpaired *t*-tests.

NPPCs, derived from the notochord in the early developmental stage, possess stem/progenitor cell properties and are crucial for cell replenishment and tissue repair in LD (Chen et al., 2024; Gan et al., 2021; Gao et al., 2022; Sakai et al., 2012; Tu et al., 2022). To explore the developmental trajectories among NP cell types, we performed pseudotime analysis on all chondrocyte populations within the NP. As expected, NPPCs were located at the starting point of the pseudotime trajectory, differentiating into two distinct lineages, termed state 2 and state 3 (Fig. 3A). Along the trajectory, we observed progressive downregulation of NPPC marker genes, *IGF1* and *PRRX1*, providing molecular evidence supporting the proposed differentiation pathway from NPPCs to NPCs (Fig. S2B and S2C).

Notably, we observed a reduction in NPPC proportions at the initial pseudotime stage (state 1) in both aged and herniated NP samples (Fig. 3B and 3C). This was confirmed by a decrease in PRRX1-positive NPPCs under aging and herniation conditions, indicating a depletion of progenitor cells (Fig. 3D). Consistent with this, genes highly expressed in state 1, such as those involved in regulation of stem cell differentiation, regulation of growth, and cell cycle, were downregulated in ON or YH NPPC compared to the YN group (Fig. 3E and 3H). These findings support the notion of NPPC exhaustion in LD aging and herniation.

Chondrocyte state 2, predominantly composed of NPC 1, was uniformly distributed in ON and YH groups (Fig. 3A–C). Dynamic gene expression analysis from state 1 (NPPC) to state 2 (mainly NPC 1) revealed upregulation of genes related to ossification and degradation of ECM (Fig. 3E). This pattern suggests that the emergence of NPC 1 may represent a calcification-related cellular response associated with both disease progression and aging. Enhanced ossification in aged NP, as shown by Alizarin Red staining, supported this hypothesis (Fig. 3F). A similar trend was also observed in herniated NP samples (Fig. 3F).

NPC 2 were predominantly present at the end of state 3 and mainly distributed in the ON group (Fig. 3A–C). Gene expression profiling along the pseudotime trajectory from state 1 (NPPC) to state 3 (mainly NPC 2) showed upregulation of genes related to ferroptosis, cellular response to oxidative stress, and cellular senescence (Fig. 3E). This indicates a potential maladaptive differentiation from NPPC and a dysfunctional characteristic of NPC 2 in ON. An increase in 4-HNE-positive cells, indicative of lipid oxidative damage (Li et al., 2022), was uniquely observed in ON group (Fig. 3G).

### *NFATC2* as a central regulator of gene expression changes in aged and herniated NPPCs

Since NPPC exhaustion is a common feature in both aging and herniated conditions, and given the potential for biased chondrocyte differentiation stemming from NPPC changes, we analyzed gene expression alterations in NPPC during aging or herniation. We found that the gene set score for NPPC marker genes was reduced, suggesting a loss of cell identity in both ON and YH NPPC (Fig. 3H and 3I). Concurrently, genes associated with cellular senescence was upregulated, while those related to the cell cycle were downregulated under both conditions (Fig. 3J and 3K), consistent with the observed accumulation of senescent cells in the NP of both conditions (Fig. 1G).

To identify pro-senescence factors in NPPC, we conducted an upstream regulon analysis across all chondrocyte cell types and identified shared upstream regulons for DEGs in both conditions (Table S4). Notably, *NFATC2* emerged as a prominent transcription factor for NPPC and NPC DEGs in both conditions, in line with its known role in adult stem cell proliferation and differentiation (Khodeer and Era, 2017; Ranger et al., 2000) (Fig. 3L). Further analysis revealed potential downstream targets of *NFATC2*, including *BMP6*, a gene critical in osteogenic progression (Novak et al., 2022; Wang et al., 2020b) (Fig. S2D). Immunohistochemical assessment confirmed elevated expression of NFAT1 (encoded by *NFATC2*) in NP samples from both aged and herniated LD (Fig. 3M), highlighting the potential regulatory role of *NFATC2* in NPPC.

### NFAT1 expression correlates with senescence in NPPCs

**Figure 4.**
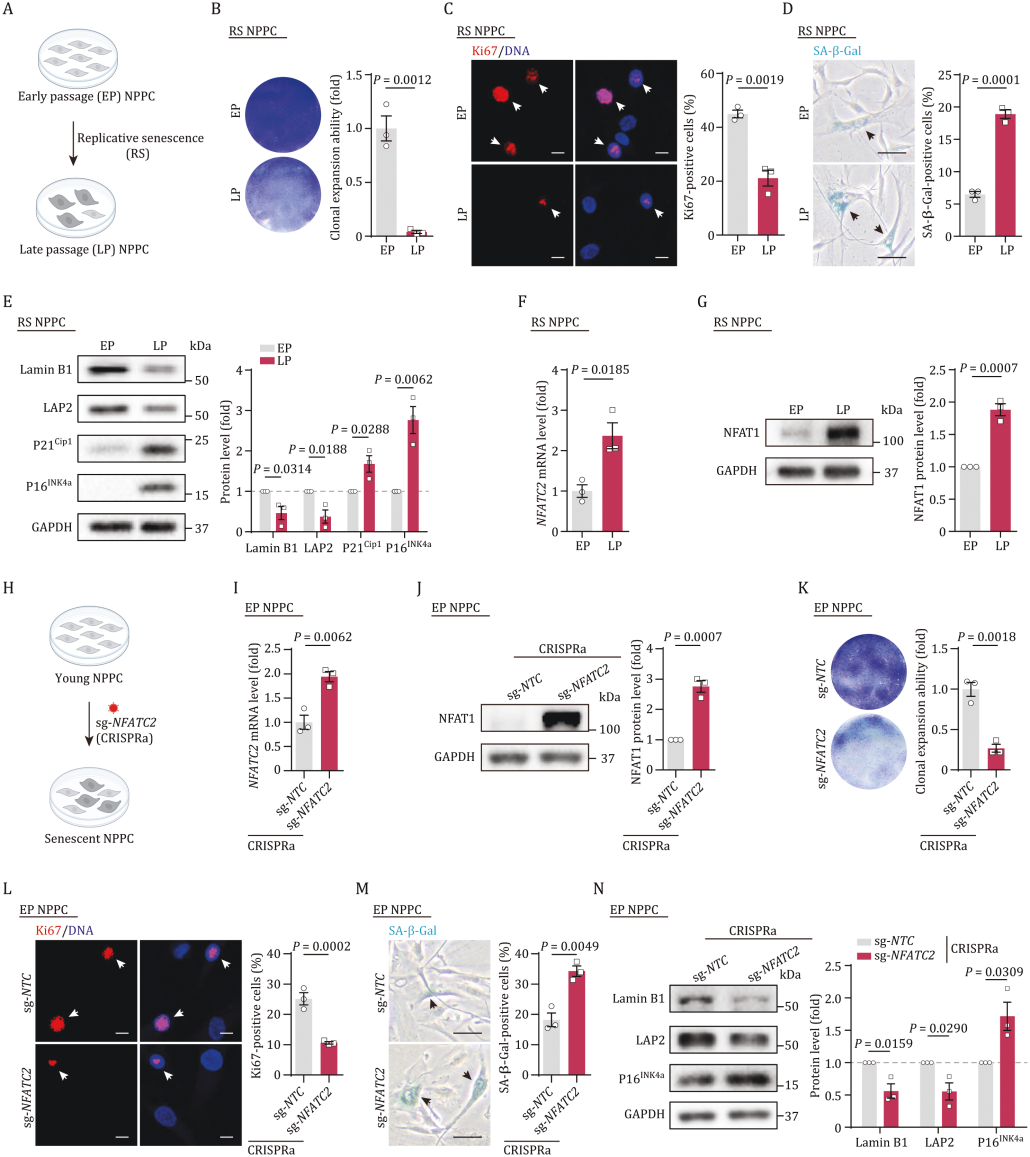
**Elevated NFAT1 reinforces the senescence of NPPCs.** (A) Schematic diagram of the model of NPPC replicative senescence. (B) Clonal expansion ability analysis in EP (Passage 4) and LP (Passage 12) NPPCs. Left, representative images. Right, the cell density is shown as the mean ± SEM. (C) Immunofluorescence of Ki67 in EP and LP NPPCs. Left, representative images, scale bars, 10 μm. Right, the percentage of Ki67-positive cells is shown as the mean ± SEM. The arrows indicate Ki67-positive cells. (D) SA-β-Gal staining in EP and LP NPPCs. Left, representative images, scale bars, 25 μm. Right, the percentage of SA-β-Gal-positive cells is shown as the mean ± SEM. The arrows indicate SA-β-Gal-positive cells. (E) Western blot analysis of P16^INK4a^, P21^Cip1^, LAP2 and Lamin B1 in EP and LP NPPCs. GAPDH was used as loading control. Data are presented as the mean ± SEM. (F) RT-qPCR analysis for the mRNA expression level of *NFATC2* in the EP and LP NPPCs. Data are presented as the mean ± SEM. (G) Western blot analysis of NFAT1 in the EP and LP NPPCs. GAPDH was used as loading control. Data are presented as the mean ± SEM. (H) Schematic diagram of the CRISPR-mediated activation (CRISPRa) of *NFATC2* in EP NPPCs. (I) RT-qPCR analysis for the mRNA expression level of *NFATC2* in the EP NPPCs after CRISPR-mediated activation of *NFATC2*. Data are presented as the mean ± SEM. (J) Western blot analysis of NFAT1 in EP NPPCs after CRISPR-mediated activation of *NFATC2*. GAPDH was used as a loading control. Data are presented as the mean ± SEM. (K) Clonal expansion ability analysis in EP NPPCs after CRISPR-mediated activation of *NFATC2*. Left, representative images. Right, the cell density is shown as the mean ± SEM. (L) Immunofluorescence of Ki67 in EP NPPCs after CRISPR-mediated activation of *NFATC2*. Left, representative images, scale bars, 10 μm. Right, the percentage of Ki67-positive cells is shown as the mean ± SEM. The arrows indicate Ki67-positive cells. (M) SA-β-Gal staining in EP NPPCs after CRISPR-mediated activation of *NFATC2*. Left, representative images, scale bars, 25 μm. Right, the percentage of SA-β-Gal-positive cells is shown as the mean ± SEM. The arrows indicate SA-β-Gal-positive cells. (N) Western blot analysis of P16^INK4a^, LAP2 and Lamin B1 in EP NPPCs after CRISPR-mediated activation of *NFATC2*. GAPDH was used as loading control. Data are presented as the mean ± SEM. *n* = 3 biological repeats per group. Statistical significance was assessed using two-tailed Student’s unpaired *t*-tests. The figures 4A and 4H were created with BioRender.com.

To delve into the molecular mechanisms behind NPPC senescence, we isolated NPPCs from human NP tissue for primary cell culture (Figs. 4A and S4A). These cells exhibited typical NPPC marker and were passageable, but reached cell cycle arrest at late passage (LP) (Fig. S4B). A comparative analysis between early passage (EP) and LP NPPCs revealed hallmarks of cellular senescence in LP NPPCs, including reduced proliferation capacity, indicated by lower clonal expansion and decreased Ki67-positive cells, alongside increased SA-β-Gal-positive cells (Fig. 4B–D). Additionally, LP NPPCs showed higher levels of P21^Cip1^ and P16^INK4a^, and lower levels of LAP2 and Lamin B1 (Fig. 4E).

Concomitant with the impaired proliferative ability, the chondrogenic differentiation potential of LP NPPCs was also compromised, as evidenced by the smaller size of differentiated chondrocyte spheres from LP NPPCs compared to EP NPPCs (Fig. S3A). RT-qPCR and Western blot analysis showed approximately a two-fold increase in *NFATC2* mRNA and protein levels in senescent NPPCs, aligning with our observations in aged and herniated NP tissues (Figs. 3M, 4F and 4G).

Furthermore, we isolated primary NPPCs from NP tissues of young and old individuals for detailed analysis (Fig. S4A). Consistent with the replicative senescence observed in NPPCs, physiologically aged NPPCs displayed similar senescence phenotypes, including retarded cellular growth, impaired proliferation, and elevated senescence markers (Fig. S4C–E). Western blot confirmed increases in P21^Cip1^ and P16^INK4a^, and decreases in LAP2 and Lamin B1 (Fig. S4F). Notably, NFAT1 expression was upregulated in aged NPPCs (Fig. S4G).

### Elevated NFAT1 promotes senescence in NPPCs

To elucidate the role of NFAT1 in NPPC senescence, we employed CRISPR-based activation (CRISPRa) to enhance *NFATC2* expression in primary NPPCs (Fig. 4H–J). Activation of *NFATC2* led to premature senescence in NPPCs, characterized by diminished clonal formation, reduced Ki67 staining, and an upsurge in SA-β-Gal-positive cells (Fig. 4K–M). The increased expression of *NFATC2* resulted in increased P21^Cip1^ and P16^INK4a^ expression, and decreased LAP2 and Lamin B1 levels (Figs. 4N and S3B).

**Figure 5.**
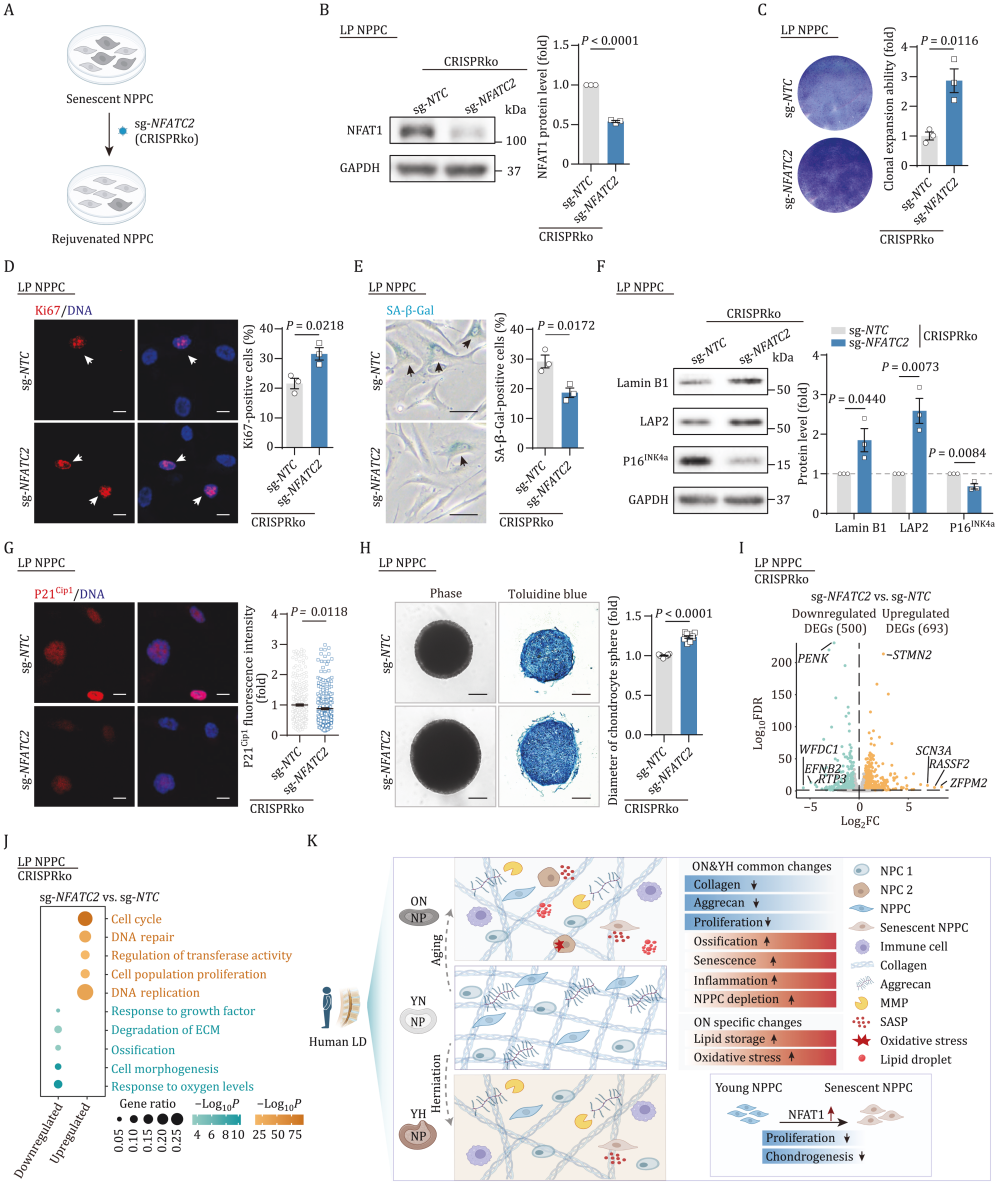
**Knockout of *NFATC2* delays the senescence of NPPCs.** (A) Schematic diagram illustrating the CRISPR-mediated knockout (CRISPRko) of *NFATC2* in LP NPPCs. (B) Western blot analysis of the expression of NFAT1 in LP NPPCs after knockout of *NFATC2*. GAPDH was used as a loading control. Data are presented as the mean ± SEM. *n* = 3 biological repeats per group. (C) Clonal expansion ability analysis in LP NPPCs after knockout of *NFATC2*. Left, representative images. Right, the cell density is shown as the mean ± SEM. *n* = 3 biological repeats per group. (D) Immunofluorescence of Ki67 in LP NPPCs after knockout of *NFATC2*. Left, representative images, scale bars, 10 μm. Right, the percentage of Ki67-positive cells is shown as the mean ± SEM. *n* = 3 biological repeats per group. The arrows indicate Ki67-positive cells. (E) SA-β-Gal staining in LP NPPCs after knockout of *NFATC2*. Left, representative images, scale bars, 25 μm. Right, the percentage of SA-β-Gal-positive cells is shown as the mean ± SEM. *n* = 3 biological repeats per group. The arrows indicate SA-β-Gal-positive cells. (F) Western blot analysis of P16^INK4a^, LAP2 and Lamin B1 in LP NPPCs after knockout of *NFATC2*. GAPDH was used as loading control. Data are presented as the mean ± SEM. *n* = 3 biological repeats per group. (G) Immunofluorescence of P21^Cip1^ in the LP NPPCs after knockout of *NFATC2*. Left, representative images, scale bars, 10 μm. Right, the fluorescence intensity of P21^Cip1^ is shown as the mean ± SEM. *n* = 300 cells per group. (H) Chondrogenesis capacity analysis in LP NPPCs after knockout of *NFATC2*. Left, representative images, scale bars, 100 μm. Right, diameter of the chondrocyte sphere is shown as the mean ± SEM. *n* = 10 biological repeats per group. (I) Volcano plot showing the DEGs in LP NPPCs after knockout of *NFATC2*. Genes with top log_2_FC value and log_10_FDR value were labeled. (J) Functional enrichment for DEGs of LP NPPCs after knockout of *NFATC2* compared to its control. The size of the dots represents the ratio of DEGs in the enriched gene sets, the depth of color of the dots represents the −log_10_*P* of enrichment. (K) Schematic chart showing the histological changes and transcriptomic signatures of human NP during LD aging and herniation. Statistical significance was assessed using two-tailed Student’s unpaired *t*-tests. The figures 5A and 5K were created with BioRender.com.

In contrast, CRISPR-mediated knockout (CRISPRko) of *NFATC2* bolstered cellular self-renewal, evidenced by expanded cloning efficiency and elevated Ki67 positivity (Fig. 5A–D). This genetic intervention also alleviated cellular senescence, as indicated by a reduction in SA-β-Gal-positive cells and downregulation of P21^Cip1^ and P16^INK4a^, concurrent with upregulation of LAP2 and Lamin B1 (Fig. 5E–G). While knockout of *NFATC2* in NPPC had no impact on its osteogenic or adipogenic differentiation capacity, it enhanced its chondrogenic potential, as indicated by enlarged chondrocyte sphere diameters (Figs. 5H, S3C and S3D). RNA sequencing (RNA-seq) analysis disclosed an upregulation of DEGs related to cell cycle progression and a downregulation of DEGs associated with ECM breakdown (Fig. 5I and 5J; Table S6). These transcriptomic shifts align with the revitalized self-renewal and differentiation potential of NPPCs.

Altogether, these results confirm that NFAT1 is a pro-senescence factor in NPPCs, where its overexpression exacerbates cellular senescence, hindering self-renewal and chondrogenesis, potentially driving the aging and herniation processes of the LD (Fig. 5K).

## Discussion

In this study, we conducted a comparative analysis of the phenotypic characteristics of the NP in aged and herniated human LDs. Our findings revealed common features, such as ECM degradation, an increased proportion of senescent cells, and heightened inflammation in both conditions. By leveraging the cellular heterogeneity of NP tissues elucidated through single-nucleus RNA sequencing, we focused our analysis on chondrocytes and their progenitors, which demonstrated the most pronounced changes in gene expression during aging and herniation. A key finding was the depletion and senescence of NPPCs, a notable alteration observed consistently in aged and herniated NP tissues. This phenomenon appears to be a critical factor regulating LD aging and herniation. Furthermore, we identified increased expression of NFAT1 as a driving force for NPPC senescence, potentially representing a common underlying mechanism for LD aging and herniation. These insights into the cellular and molecular mechanisms underlying LD aging and herniation could inform the development of targeted therapeutic strategies for the treatment of intervertebral disc disorders.

Both structural and functional declines in the LD are observed in aging and herniation, yet most studies treat these conditions as a single entity of degenerated LDs, often including elderly individuals with disc herniation without distinguishing the phenotypic and molecular differences between aging and herniation (Cherif et al., 2022; Wang et al., 2023). We present the first comparative single-nucleus transcriptomic analysis of LD aging and herniation, identifying biological process changes associated with each condition. Notably, lipid accumulation was exclusively elevated in the aging group, suggesting this process may be primarily attributed to aging. This observation underscores the necessity for future investigations to delineate molecular changes and biological processes unique to either pathological disorder or physiological aging. Such differentiation could facilitate a more nuanced understanding of LD herniation and more efficacious therapeutic interventions.

While prior single-cell analyses have demonstrated NPPC exhaustion in herniated disc tissues (Gao et al., 2022; Ma et al., 2023; Swahn et al., 2024; Tan et al., 2024; Tu et al., 2022; Wang et al., 2023), the age-related changes in NPPC remain incompletely understood. Our single-nucleus transcriptomic data demonstrates that the senescence of NPPCs may serve as a major factor contributing to their depletion in both LD aging and herniation. Pseudotime analysis indicated that NPPCs gradually transition into different NPC states during these processes. Building upon previous findings of enriched calcifying chondrocytes in herniated disc tissues (Lu and Zheng, 2023), our analysis revealed that calcification represents a shared characteristic between aging and herniation. This was evidenced by the transition of NPPC to NPC 1, which displayed expression patterns of ossification-associated genes consistent with the phenotype of calcifying chondrocytes. Alizarin Red staining validation confirmed enhanced calcification in aged and herniated NP samples, demonstrating that calcification serves as a common pathological hallmark in both LD aging and herniation. Notably, we identified a previously unrecognized cell state, NPC 2, which was exclusively found in NP samples from aging individuals. These cells displayed elevated lipid synthesis and storage, heightened TGFβ receptor signaling, and potential susceptibility to ferroptosis and senescence, as indicated by their gene expression patterns. This biased differentiation may result from a loss of NPPC cell identity, necessitating further experiments to explore the relationship between cell senescence and the loss of NPPC identity. In line with this possibility, we observed a compromised chondrogenic capability in senescent NPPCs. Our study elucidates the changes in chondrocytes and their progenitors during LD herniation and aging, highlighting how the senescence of NPPCs and the resulting functional decline contribute to both LD aging and herniation.

NFAT1, a member of the nuclear factor of activated T cells (NFAT) family, is a calcium-responsive protein extensively characterized for its roles in immune responses (Martinez et al., 2015; Xanthoudakis et al., 1996). Its activation involves calcineurin-mediated dephosphorylation, leading to nuclear translocation and transcriptional regulation (Rao et al., 1997). NFAT1 has also been implicated in bone development and homeostatic maintenance. For instance, mice overexpressing NFAT1 demonstrate osteoporosis and a reduction in bone volume, while germline deletion of *Nfatc2* has been associated with osteoarthritis (Greenblatt et al., 2013; Zanotti and Canalis, 2015). In our study, we found that NFAT1 is consistently upregulated in both human LD aging and herniation, with its increased expression identified as a potential driver of NPPC senescence. *COL6A1,* a well-recognized driver and biomarker of fibrosis was identified as a target gene of *NFATC2* (Bonnemann, 2011). Our data identified the increase of *COL6A1* in both LD aging and herniation, aligning with the fibrosis observed in histological assessments.

Given that *NFATC2* is a key driver of senescence in NPPCs and may be an important cause for both LD aging and herniation. In the future, experiments such as using gene editing technology to knockout *NFATC2* in aged or herniated animal models to observe whether it can delay or prevent the occurrence of LD aging and herniation diseases are warranted (Doudna, 2020; Peng et al., 2024; Yang et al., 2023; Zhang et al., 2018). Additionally, we have found that young NPPCs possess higher proliferative and chondrogenic differentiation capabilities. With advancements in stem cell transplantation technology (Choi et al., 2015; Trounson and McDonald, 2015; Zhu et al., 2023), we may attempt to maintain or restore the function of aged or herniated discs by transplanting young NPPCs or NPPCs with *NFATC2* knockout. Furthermore, we can explore small molecules that inhibit *NFATC2* expression to determine if they can delay the progression of LD aging and disease.

In summary, we systematically investigated the phenotypic and molecular changes in the NP tissue of aged and herniated LDs, providing valuable insights into the key alterations and molecular mechanisms involved in NP aging and herniation. Our findings suggest that NPPCs play a critical role in LD aging and herniation, with NFAT1 identified as a critical transcription factor regulating NPPC senescence. This discovery offers potential therapeutic targets for the treatment of aging related degenerative disc diseases.

## Materials and methods

### Patients and specimens

During the sample collection process, we grouped the samples based on age and Pfirrmann grading of LD degeneration. Young NP samples (YN group, *n* = 6, aged 15–38) were diagnosed with Pfirrmann grade II or lower through magnetic resonance imaging (MRI), and old NP samples (ON group, *n* = 11, aged 60–80) were diagnosed with Pfirrmann grade II or lower through MRI. Young herniated NP samples (YH group, *n* = 11, aged 15–38) were diagnosed with Pfirrmann grade IV or higher through MRI (see Table S1). The Pfirrmann grading is a classic method for assessing LD degeneration using MRI imaging (Pfirrmann et al., 2001), with grades II or lower indicating a healthier LD, which we used as the young control group or old group, and grades IV or higher indicating a typical LD herniation disease state. The YN samples were obtained from young patients with scoliosis requiring corrective surgery, who had otherwise healthy discs. YH samples were sourced from young patients diagnosed with LD herniation. ON samples were acquired from elderly patients presenting with lumbar spinal stenosis. All NP tissues were surgically excised due to the abovementioned lumbar disc pathology. Exclusion criteria encompassed lumbar spine trauma, infection, tuberculosis, tumors, and autoimmune diseases. This study received approval from the Human Subjects Institutional Review Board at Peking University First Hospital (Approval No.: 2021-KY-252). A single investigator conducted all sample collections to ensure consistency.

In the single-nucleus RNA sequencing, we took the NP tissues of the same size from 3 to 8 individuals. These tissues were then pooled into different groups, and nuclei were isolated for single-nucleus RNA sequencing. For the YN group, NP tissues from 3 individuals were mixed. For the ON group and the YH group, tissues from 8 individuals were mixed, respectively. And the individual information was listed in Table S1.

In the experimental validation, in order to ensure the consistency of the number of individuals among each group, we randomly selected three individuals from each group for subsequent experimental validation, and listed the individual information in Table S1.

### Hematoxylin and eosin (H&E) staining

Paraffin-embedded NP tissue sections were first deparaffinized by soaking in xylene twice for 10 min each, followed by rehydration using a gradient of ethanol from high to low concentrations along with water. Subsequently, the rehydrated sections were immersed in hematoxylin for 3 min for DNA staining, rinsed in water to remove excess color, and finally counterstained with eosin. After dehydration with increasing concentrations of ethanol and cleaning with xylene, the slides were mounted. Images were captured using a microscope (Nikon, CV2000).

### Masson-trichrome staining

Masson-trichrome staining was performed as previously described (Liu et al., 2024; Wang et al., 2020a). Paraffin sections of NP tissue were deparaffinized by soaking in xylene and rehydrated with ethanol series and water. Masson-trichrome staining was performed according to the manufacturer’s instructions (Solarbio). The images were collected using microscope (Nikon, CV2000) and analyzed by the ImageJ.

### SA-β-Gal staining

The SA-β-Gal staining experiment was carried out according to the methods that have been previously described (Huang et al., 2024; Ma et al., 2024). In brief, the frozen sections with a thickness of 10 μm of the human NP tissue or cultured NPPCs were fixed with a solution containing formaldehyde and glutaraldehyde at room temperature for 5–10 min. Subsequently, the samples were incubated with a staining solution containing 20 mg/mL X-Gal at 37°C. For SA-β-Gal staining of collected tissues, we took six images per individual using a Nikon microscope (CV2000) with the same parameters (exposure time and light intensity) under a 20× objective. The ImageJ was used to analyze the SA-β-Gal-positive area, and the SA-β-Gal-positive area value for each individual was calculated by averaging the signal area across all fields of vision. For cellular SA-β-Gal staining, we took an equal number of cell images for quantification in each biological replicate, with the number of cells per biological replicate exceeding 300, and we have calculated the proportion of SA-β-Gal positive cells.

### Oil Red O staining

The Oil Red O staining procedure was carried out according to the method previously described (Ma et al., 2020; Yang et al., 2024a). After preparing the Oil Red O working solution (Sigma-Aldrich) in accordance with the manufacturer’s instructions and filtering it through a 0.45-μm filter, the OCT-embedded NP tissue sections were stained by soaking them in the filtered staining solution for 15 min. Following staining, rinse the slides with clean water and counterstain with hematoxylin for DNA staining. Samples were subsequently visualized using microscopy (Nikon, CV2000), and quantitative analysis was performed using ImageJ software.

### Immunohistochemistry

The paraffin sections of NP tissue were deparaffinized with fresh xylene and rehydrated with a series of ethanol and water. The sections were then immersed in preheated citrate buffer (pH = 6.0) and further heated in a microwave for 20 min for antigen retrieval. After cooling to room temperature, the sections were washed several times with PBS. Subsequently, the paraffin or OCT sections were permeabilized in a solution of 0.4% Triton X-100 for 30 min. They were then incubated in a 3% H_2_O_2_ solution for 20 min. Finally, the sections were blocked with 5% donkey serum (Jackson ImmunoResearch) for 1 h at room temperature. After blocking, diluted primary antibodies were applied to the slides and incubated overnight at 4°C. The next day, the primary antibodies were removed, and the sections were washed several times with PBS before incubation with secondary antibodies. Subsequently, colorimetric detection was performed using DAB, and the sections were counterstained with hematoxylin for DNA staining. Finally, the stained sections were dehydrated through a graded series of ethanol and cleared with xylene before being mounted with neutral gum. Immunohistochemistry staining images were captured using a Nikon microscope (CV2000) with the same parameters (exposure time and light intensity) under a 20× objective, and the same number of fields of vision (5 or more) were taken for each individual. Subsequently, the same number of images for each individual were used to quantify the positive area or intensity of the staining signal. We used ImageJ with the same parameters to obtain the area or grayscale values of the positive signal. The signal intensity or area value for each individual was obtained by averaging the signal intensity or area across all fields of vision.

The following antibodies were employed in this study: anti-Collagen II (Abcam, ab34712), anti-MMP9 (Abcam, ab38898), anti-Ki67 (ZSGB-Bio, ZM-0166), anti-P21^Cip1^ (Cell Signaling Technology, 2947S), anti-HERV-K-env (Austral Biologicals, HERM-1811-5), anti-IL-1β (Santa Cruz Biotechnology, sc-52012), anti-CD68 (Abcam, ab955), anti-Aggrecan (Abcam, ab3778), anti-PRRX1 (Abcam, ab211292), and anti-NFAT1 (Santa Cruz Biotechnology, sc-7296).

Immunohistochemistry staining for HERV-K-env was performed on paraffin-embedded tissues, while the remaining Immunohistochemistry stainings were done on OCT-embedded tissues.

### Isolation of NPPCs and cell culture

NPPCs were isolated from NP tissue following previously established protocols (Otani et al., 2024). The procedure began with washing the NP tissue in PBS supplemented with 2% penicillin/streptomycin (Thermo Fisher Scientific). The washed tissue was then placed in a 6-well plate and incubated at 37°C for 2 h. Subsequently, 2 mL of culture medium was added to each well. The tissue was maintained in culture, with medium changed every 2 days, until NPPCs migrated out of the tissue.

The components for the culture medium used to culture NPPCs are as follows: 500 mL of MEM α basal medium (Thermo Fisher Scientific), 10% fetal bovine serum (FBS, Gemini), 0.1 mmol/L non-essential amino acids (NEAA, Gibco), 1% penicillin/streptomycin (Gibco), and 1 ng/mL FGF2 (Joint Protein Central).

The differentiation method of NPPCs into adipocytes, chondrocytes and osteoblasts was described previously (Liu et al., 2014). The adipogenic, chondrogenic, and osteogenic potentials of NPPC were identified by Oil Red O staining (Sigma-Aldrich), toluidine blue staining (Sigma-Aldrich), and Von Kossa staining (Genmed Scientifics), respectively.

### Plasmid construction

By cloning sgRNAs targeting *NFATC2* into the lentiCRISPR v2 or lentiSAMv2 plasmids, the lentivirus vectors for CRISPRko or CRISPRa for knockout or activation of *NFATC2* are constructed. The sequences of sg-*NFATC2* are listed in Table S5.

### Clonal expansion assay

1 mL of culture medium containing 3,000 cells was added to each well of a 12-well plate and cultured until the cell density of a group of cells exceeded 90% confluence. The cells were then washed several times with PBS, and fixed with 4% paraformaldehyde (PFA) at room temperature for 30 min. Subsequently, 1 mL of 0.2% crystal violet solution (Biohao) was added for staining for 1 h before the cells were rinsed with water, and the images were scanned using Epson Perfection V370 Photo (EPSON). Quantitative analysis of cell density was conducted using ImageJ software.

### Immunofluorescence

OCT-embedded NP tissue sections were washed several times with fresh PBS, then fixed with 4% PFA for 10 min, and subsequently permeabilized in a PBS solution containing 0.4% Triton X-100 for 30 min. Similarly, cells were treated for 15 min each in 4% PFA and PBS solutions containing 0.4% Triton X-100 for fixation and permeabilization, respectively. After that, the NP tissue sections or cells were treated with 5% donkey serum at room temperature for 1 h and incubated with diluted primary antibodies overnight at 4°C. The samples were then washed three times with PBS and incubated with diluted secondary antibodies in the dark at room temperature for 1 h. Afterwards, the DNA was labeled with Hoechst 33342 (Thermo Fisher Scientific) before the samples were mounted. Images were captured using a microscope. The following antibodies were employed in this study: anti-Ki67 (ZSGB-Bio, ZM-0166), anti-Ki67 (Abcam, ab15580), anti-P21^Cip1^ (Cell Signaling Technology, 2947S), anti-PRRX1 (Abcam, ab211292), anti-CD45 (Abcam, ab8216), and anti-4-HNE (ABclonal, A26085).

### Western blot (WB) analysis

Cell protein samples, lysed using 1× SDS lysis buffer (50 mmol/L Tris-HCl, pH 6.8, 10% glycerol, 2% SDS, and 2% β-mercaptoethanol) and denatured at 105°C for 10 min, were quantified using the BCA protein assay kit (Beijing Dingguo Changsheng Biotechnology). Then the protein samples underwent SDS-PAGE separation and were electrotransferred onto a 0.2-μm PVDF membrane (Millipore). The PVDF membrane was blocked with 5% (*w*/*v*) nonfat dry milk on a shaker at room temperature for 1 h and then incubated with diluted primary antibodies overnight at 4°C. Subsequently, the PVDF membrane was incubated with HRP-conjugated secondary antibodies at room temperature for 1 h. Finally, visualization was performed using a ChemiDoc XRS+ system (Bio-Rad), and the data were analyzed with ImageJ software. The following antibodies were employed in this study: anti-P16^INK4a^ (BD, 550834), anti-LAP2 (BD, 611000), anti-P21^Cip1^ (Cell Signaling Technology, 2947s), anti-Lamin B1 (Abcam, ab16048), anti-NFAT1 (Cell Signaling Technology, 5861T), and anti-GAPDH (Santa Cruz Biotechnology, sc-365062).

### Real-time quantitative PCR assay

Total RNA was extracted using the TRIzol reagent kit reagent (Invitrogen) according to the manufacturer’s instructions. Following the manufacturer’s instructions, RNA was reverse transcribed into cDNA using GoScript Reverse Transcription System (Promega). Subsequently, the primers were mixed with THUNDERBIRD SYBR qPCR Mix (Toyobo), and the cDNA samples along with the primers were added to a 384-well plate (Bio-Rad) for RT-qPCR on the QuantStudio™ 5 Real-Time PCR System (Applied Biosystems). The relative transcript expression level of all gene was normalized to the GAPDH transcript. The primer sequences are listed in Table S5.

### Nuclei isolation and single-nucleus RNA sequencing on the 10x Genomics platform

Following the previously published method (Sun et al., 2023; Yang et al., 2024b), all sample handling steps were conducted on ice for the isolation of nuclei from human NP tissues. Briefly, the frozen tissues were first grounded using a pestle and mortar, and then the samples were solubilized in 1.5 mL of lysis buffer prepared with nuclease-free water, which contained 250 mmol/L sucrose, 1 μmol/L dithiothreitol, 5 mmol/L MgCl_2_, 25 mmol/L KCl, 1× protease inhibitor, 0.4 U/μL RNaseIn, 10 mmol/L Tris buffer, 0.1% Triton X-100, and 0.2 U/μL Superasin. The sample solution was then filtered through a 40 μm filter and transferred into 1.5 mL centrifuge tubes, which were centrifuged at 500 ×*g* for 8 min at 4°C. The nuclei were stained with acridine orange and propidium iodide, and double-positive nuclei were sorted using FACS (BD Influx). Nuclei from the same group of NP tissues were pooled and subjected to single-nucleus capture using the 10x Genomics Single-Cell 3′ system. Approximately 9,000 nuclei were captured per sample following the standard 10× capture and library preparation protocol (10x Genomics), and then sequenced on a NovaSeq 6000 sequencing system (Illumina, 20012866).

### Bulk RNA-seq data processing

Total RNA was extracted using the TRIzol reagent kit reagent (Invitrogen) according to the manufacturer’s instructions, and the RNA was sequenced with HiSeq-PE150. We used fastp (version 0.23.2) software for quality control, adapter trimming, and quality filtering of raw reads. As for mapping the trimmed reads to the Homo sapiens GRCh37.87 genome, HISAT2 (version 2.0.4) (Kim et al., 2015) was used. Then the generated sam files were converted to bam files through SAMtools (version 1.6). The read count of each gene was calculated through the featureCounts (version 2.0.3) software. We used the R package DESeq2 (version 1.2.4) (Love et al., 2014) to identify DEGs between groups, with the threshold cutoff values of adjusted *P* value < 0.05 and |Log_2_FC| ≥ 0.5.

### Processing of raw data from single-nucleus RNA sequencing

Single-cell capture and transcriptome library were established using 10x Genomics platform, Chromium Single-Cell 3′ Gel Bead and Library V2 Kit, and sequenced on NovaSeq 6000 sequencing platform (Illumina 20012866) to obtain single-nucleus transcriptome data. Cell Ranger (4.0.0) was further used to create pre-mRNA reference of Homo sapiens GRCh37.87 genome, and the gene expression matrix for downstream analysis was calculated by the “count” function and default parameters. CellBender software (0.2.0) (Fleming et al., 2023) was used to eliminate possible background RNA bias using default parameters. Downstream analysis of filtering low-quality cells, data normalization, dimensionality reduction, and clustering were under the version of Seurat (4.0.2) (Hao et al., 2021). Cells with a gene number < 200 or a mitochondrial gene ratio > 5% were excluded as low-quality cells. DoubletFinder (2.0.3) software (McGinnis et al., 2019) was used to detect and delete possible twin data in the technical artifact. After the expression matrix of each sample was normalized and scaled by using the “SCTransfrom” function, the anchors and features for downstream integration were selected by using the “PrepSCTIntegratio” and “FindIntegrationAnchors” functions. All valid samples were integrated by using the “IntegrateData” function, and scaled by using the “ScaleData” function. After the above data integration and scaling, principal component analysis (PCA) and clustering were performed by using the “RunPCA” and “FindCluster” functions. Then UMAP dimensionality reduction was performed by using the “RunUMAP” function. Cell clustering was performed by using the “FindNeighbors” and “FindClusters” functions. The marker genes of each cluster were calculated by using the “FindAllMarkers” function (avg_log_2_FC ≥ 0.25 and *P*_val_adj < 0.05).

### Cell identity score analysis

Representative gene sets of cell identity of NPPC were calculated by using the function “FindAllMarkers” of Seurat to compute the top 10 marker genes of NPPC within chondrocytes in young group under the threshold of avg_log_2_FC ≥ 1 and *P*_val_adj < 0.05. The function “AddModuleScore” was then used to calculate the cell identity score of NPPC in YN, ON and YH groups with the top 10 marker genes.

### Differential expression analysis from single-nucleus RNA sequencing data

Seurat function “FindMarkers” with the Wilcoxon signed-rank test was used to calculate the DEGs between groups (ON/YN, YH/YN). Genes with |avg_log_2_FC| ≥ 0.75 and *P*_val_adj < 0.05 were identified as O-DEGs or H-DEGs.

### Transcriptional regulatory network analysis

We followed the R packages SCENIC (version 1.2.4) (Aibar et al., 2017) workflow with default parameters to calculate the transcriptional regulatory network of DEGs, and download TFs of hg19 genome from RcisTarget (version 1.10.0) database. Firstly, we constructed TF-genes co-expression networks through GENIE3 (version 1.12.0). Each row of the gene expression matrix represents a DEG and each column represents a nucleus of each cell type, respectively. We used RcisTarget to infer the enriched TF binding motifs and their target genes. Finally, Cytoscape (version 3.9.1) was used to visualize the TF-target genes regulatory module network.

### Pathway enrichment analysis

Enrichment analysis of Gene Ontology (GO) process and pathway were performed by clusterProfiler (4.6.0) (Wu et al., 2021) and Metascape (Zhou et al., 2019). Kappa-test scores were calculated between each of two terms selected from the enrichment results (*P* value ≤ 0.05), and set as similarity scores between terms.

### Inference of the pseudotime trajectory

Monocle2 (2.99.3) (Qiu et al., 2017) was used to study the developmental trajectories of NPPCs and NPCs. The “differentialGeneTest” function was used to select the total differential genes for the cells, and the top 200 genes ranked by q value values among these genes were selected to align the cells. “reduceDimension” is used to reduce the dimension of cells to two-dimensional space, and the “orderCells” function is used to sort the cells. The starting point is called again without modifying the root_state parameter to obtain the expected time trajectory of cell differentiation.

### Gene set score analysis

Gene sets used for score were downloaded from Kyoto Encyclopedia of Genes and Genomes (KEGG) and GO databases. The function “AddModuleScore” of Seurat was used to calculate gene sets expression scores, and score results were visualized through R package ggplot2.

### Statistical analysis

Statistical analysis was performed using GraphPad Prism 6.01. All data represent mean ± SEM. Comparisons were conducted using the two-tailed Student’s *t*-test. *P* values less than 0.05 were considered statistically significant.

## Supplementary Material

pwaf025_suppl_Supplementary_Table_S1

pwaf025_suppl_Supplementary_Table_S2

pwaf025_suppl_Supplementary_Table_S3

pwaf025_suppl_Supplementary_Table_S4

pwaf025_suppl_Supplementary_Table_S5

pwaf025_suppl_Supplementary_Table_S6

pwaf025_suppl_Supplementary_Figures_S1-S4

## Data Availability

All data associated with this study are present in the paper or the supplementary materials. The raw sequence data reported in this paper have been deposited in the Genome Sequence Archive-Human in National Genomics Data Center, China National Center for Bioinformation, with accession number HRA008829.

## Code availability

All the custom codes are available from the corresponding authors upon reasonable request.
