# Supplementary material for: Single-nucleus transcriptomics decodes the link between aging and lumbar disc herniation
Source: Protein Cell. 2025 Mar 22;16(8):667–84. doi: 10.1093/procel/pwaf025 (PMC12342153; doi:10.1093/procel/pwaf025)
Supplement: pwaf025_suppl_Supplementary_Figures_S1-S4 [file pwaf025_suppl_supplementary_figures_s1-s4.pdf]

Supplemental materials

Supplemental figure legends

Figure S1. Characterizing phenotypic changes in NP of aged and herniated human LDs.

(A) Immunohistochemistry staining of $P21^{Cip1}$ in human NP of YN, ON and YH. Left, representative images, scale bars, 100 μm and 25 μm (zoomed-in images). Right, the proportion of $P21^{Cip1}$-positive cells per field of vision is quantified. The arrows indicate $P21^{Cip1}$-positive cells.

(B) Immunohistochemistry staining of Ki67 in human NP of YN, ON and YH. Left, representative images, scale bars, 100 μm and 25 μm (zoomed-in images). Right, the proportion of Ki67-positive cells per field of vision is quantified. The arrows indicate Ki67-positive cells.

(C) Immunofluorescence staining of CD45 in human NP of YN, ON and YH. Left, representative images, scale bars, 10 μm. Right, the proportion of CD45-positive cells per field of vision is quantified. The arrows indicate CD45-positive cells.

Data are presented as the means ± SEM. n = 3 individuals per group. Statistical significance was assessed using two-tailed Student's unpaired t tests.

Figure S2. Single-nucleus transcriptomic assay of NP of aged and herniated human LD.

(A) Dot plot showing the expression level of representative marker genes across cell types. The color key from gray to red presents low to high gene expression levels. The size of dots indicates the percentage of cells with gene expression greater than zero.

(B) Featureplot showing the expression level of genes in cluster 1, 2 and 3.

(C) Temporal expression profiles of selected representative genes plotted along the pseudotime trajectory.

(D) *NFATC2*-regulated target genes in NPPCs. Node border colors indicate classification into ON or YH groups. The color key indicates $\log_2$FC in gene expression during LD aging or herniation.

Figure S3. NFAT1 induces NPPCs senescence.

(A) Chondrogenesis capacity analysis in the EP and LP NPPCs. Left, representative images, scale bars, 100 μm. Right, diameter of the chondrocyte sphere is shown as the means ± SEM. n = 11 biological repeats per group.

(B) Immunofluorescence of $P21^{Cip1}$ in the EP NPPCs after CRISPR-mediated activation of *NFATC2*. Left, representative images, scale bars, 10 μm. Right, the fluorescence intensity of $P21^{Cip1}$ is shown as the means ± SEM. n = 300 cells per group.

(C) Adipogenesis capacity analysis in the LP NPPCs after knockout of *NFATC2*. Left, representative images, scale bars, 50 μm. Right, Absorbance is shown as the means ± SEM. n = 3 biological repeats per group.

(D) Osteogenesis capacity analysis in the LP NPPCs after knockout of *NFATC2*. Left, representative images, scale bars, 50 μm. Right, Von Kossa-positive area is shown as the means ± SEM. n = 3 biological repeats per group.

Statistical significance was assessed using two-tailed Student's unpaired t tests.

Figure S4. The expression of NFAT1 is increased in physiological aged NPPCs.

(A) Schematic diagram of the isolation of NPPC from young and old NP tissues.

(B) Immunofluorescence of PRRX1 in the young and old NPPCs (Passage 4). Left, representative images, scale bars, 10 μm. Right, the percentage of PRRX1-positive cells is shown as the means ± SEM. The arrows indicate PRRX1-positive cells.

(C) Clonal expansion ability analysis in the young and old NPPCs. Left, representative images. Right, the cell density is shown as the means ± SEM.

(D) Immunofluorescence of Ki67 in the young and old NPPCs. Left, representative images, scale bars, 10 μm. Right, the percentage of Ki67-positive cells is shown as the means ± SEM. The arrows indicate Ki67-positive cells.

(E) SA-β-Gal staining in the young and old NPPCs. Left, representative images, scale bars, 25 μm. Right, the percentage of SA-β-Gal-positive cells is shown as the means ± SEM. The arrows indicate SA-β-Gal-positive cells.

(F) Western blot analysis of $P16^{INK4a}$, $P21^{Cip1}$, LAP2 and Lamin B1 in the young and old NPPCs (Passage 4). GAPDH was used as loading control. Data are presented as the means ± SEM.

(G) Western blot analysis of NFAT1 in the young and old NPPCs. GAPDH was used as loading control. Data are presented as the means ± SEM.

n = 3 biological repeats per group. Statistical significance was assessed using two-tailed Student's unpaired *t* tests.

Supplementary table legends

Table S1. Sample information used in this study.

Table S2. Marker genes of different cell types identified in single-nucleus RNA sequencing of nucleus pulposus from human young, aged and young herniated lumbar disc.

Table S3. Differentially expressed genes of single-nucleus RNA sequencing datasets of human nucleus pulposus from aged and young herniated lumbar disc.

Table S4. Core regulatory transcription factors of differentially expressed genes in human nucleus pulposus from aged and young herniated lumbar disc.

Table S5. List of primers used in this study.

Table S6. Differentially expressed genes identified by RNA-seq analysis in late passage NPPCs after lentivirus-mediated CRISPR knockout of *NFATC2* (sg-*NFATC2* vs. sg-*NTC*).

Figure S1

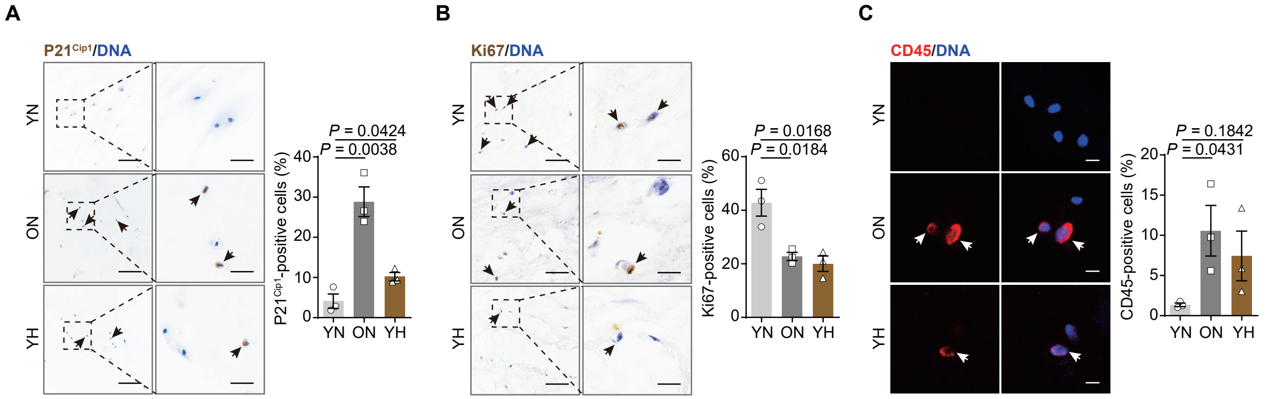

A
P21Cip1/DNA
YN
ON
YH
P = 0.0424
P = 0.0038
P21Cip1-positive cells (%)
YN ON YH
B
Ki67/DNA
P = 0.0168
P = 0.0184
Ki67-positive cells (%)
C
CD45/DNA
P = 0.1842
P = 0.0431
CD45-positive cells (%)

Figure S2

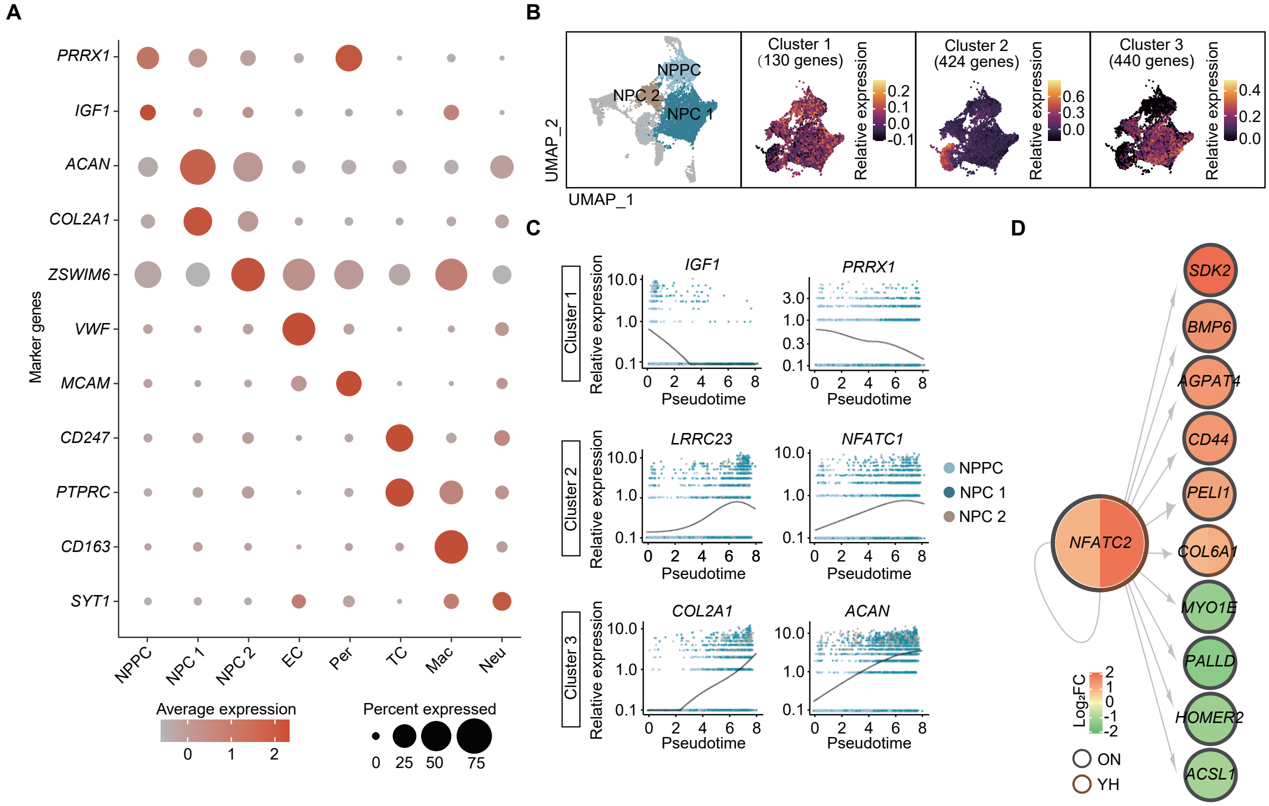

A
PRRX1
IGF1
ACAN
COL2A1
ZSWIM6
VWF
MCAM
CD247
PTPRC
CD163
SYT1
Marker genes
NPPC
NPC 1
NPC 2
EC
Per
TC
Mac
Neu
Average expression
0
1
2
Percent expressed
0
25
50
75
B
NPPC
NPC 2
NPC 1
UMAP_2
UMAP_1
Cluster 1
(130 genes)
Cluster 2
(424 genes)
Cluster 3
(440 genes)
Relative expression
C
Cluster 1
Cluster 2
Cluster 3
IGF1
PRRX1
LRRC23
NFATC1
COL2A1
ACAN
Relative expression
Pseudotime
NPPC
NPC 1
NPC 2
D
NFATC2
SDK2
BMP6
AGPAT4
CD44
PELI1
COL6A1
MYO1E
PALLD
HOMER2
ACSL1
Log₂FC
ON
YH

Figure S3

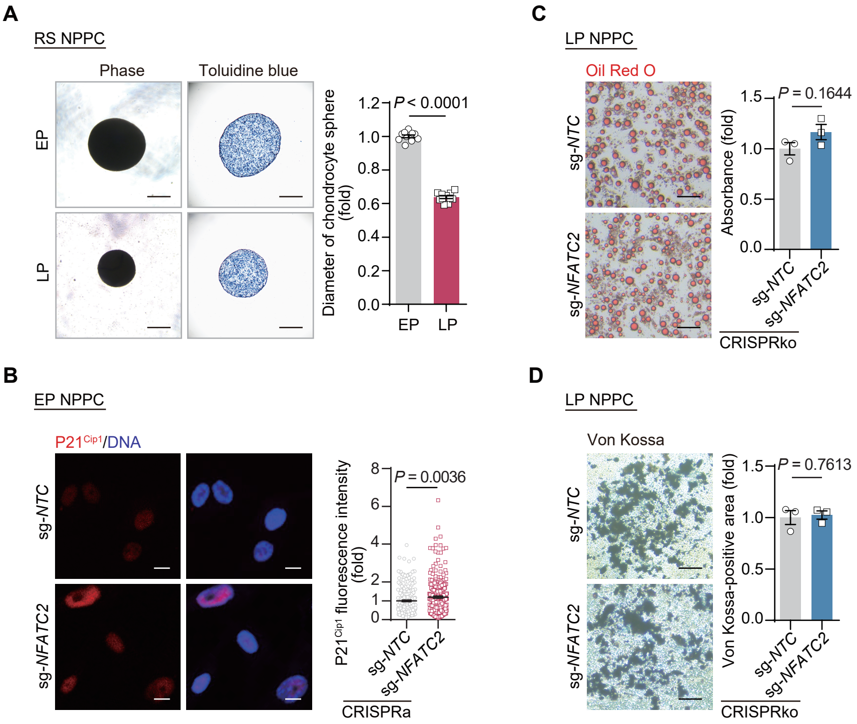
A
RS NPPC
Phase
Toluidine blue
EP
LP
Diameter of chondrocyte sphere (fold)
P < 0.0001
1.2
1.0
0.8
0.6
0.4
0.2
0.0
EP
LP
B
EP NPPC
P21Cip1/DNA
sg-NTC
sg-NFATC2
P21Cip1 fluorescence intensity (fold)
P = 0.0036
8
6
4
2
1
0
sg-NTC
sg-NFATC2
CRISPRa
C
LP NPPC
Oil Red O
sg-NTC
sg-NFATC2
Absorbance (fold)
P = 0.1644
1.5
1.0
0.5
0.0
sg-NTC
sg-NFATC2
CRISPRko
D
LP NPPC
Von Kossa
sg-NTC
sg-NFATC2
Von Kossa-positive area (fold)
P = 0.7613
1.5
1.0
0.5
0.0
sg-NTC
sg-NFATC2
CRISPRko

Figure S4

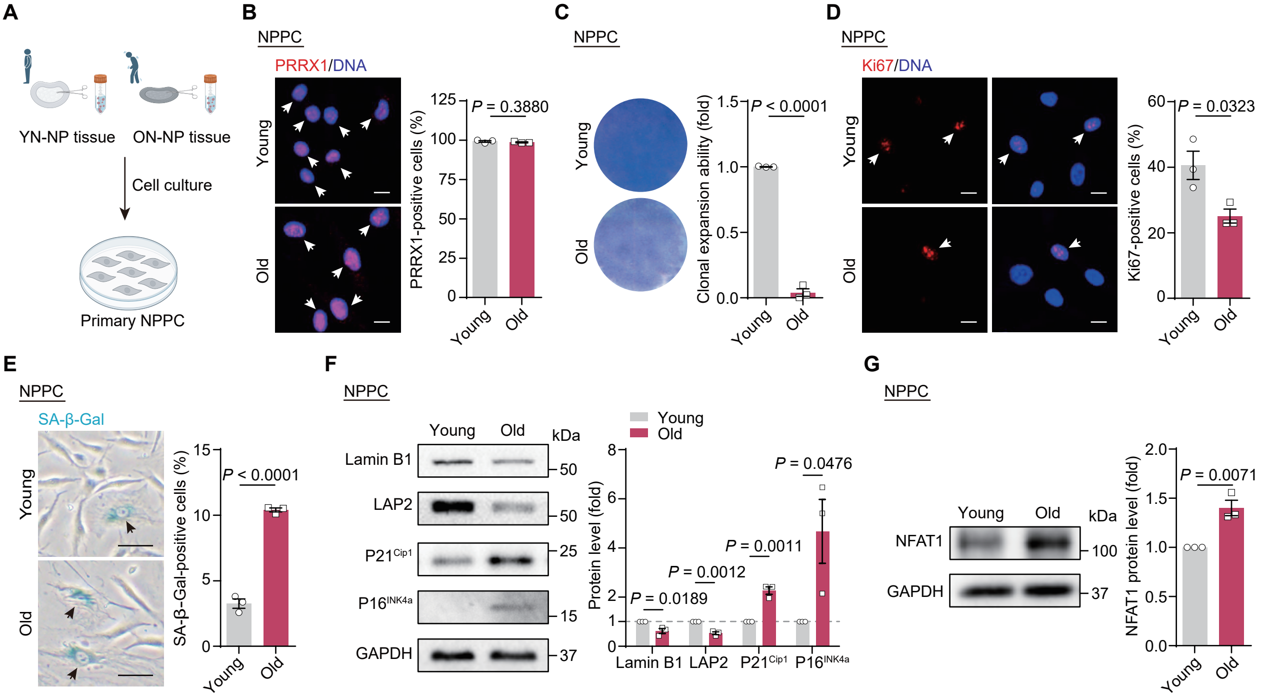
A
YN-NP tissue
ON-NP tissue
Cell culture
Primary NPPC
B
NPPC
PRRX1/DNA
Young
Old
PRRX1-positive cells (%)
P = 0.3880
C
NPPC
Young
Old
Clonal expansion ability (fold)
P < 0.0001
D
NPPC
Ki67/DNA
Young
Old
Ki67-positive cells (%)
P = 0.0323
E
NPPC
SA-β-Gal
Young
Old
SA-β-Gal-positive cells (%)
P < 0.0001
F
NPPC
Young
Old
kDa
Lamin B1
LAP2
P21Cip1
P16INK4a
GAPDH
Protein level (fold)
P = 0.0189
P = 0.0012
P = 0.0011
P = 0.0476
G
NPPC
Young
Old
kDa
NFAT1
GAPDH
NFAT1 protein level (fold)
P = 0.0071
